# pH-Dependent Effects in Nanofluidic Memristors

**DOI:** 10.1021/acs.jpclett.4c01610

**Published:** 2024-07-25

**Authors:** Sergio Portillo, José A. Manzanares, Patricio Ramirez, Juan Bisquert, Salvador Mafe, Javier Cervera

**Affiliations:** † Departament de Física de la Terra i Termodinàmica, Universitat de València, E-46100 Burjassot, Spain; ‡ Departament de Física Aplicada, Universitat Politécnica de València, E-46022 València, Spain; § Instituto de Tecnología Química, (Universitat Politècnica de València-Agencia Estatal Consejo Superior de Investigaciones Científicas), Av. dels Tarongers, 46022 València, Spain

## Abstract

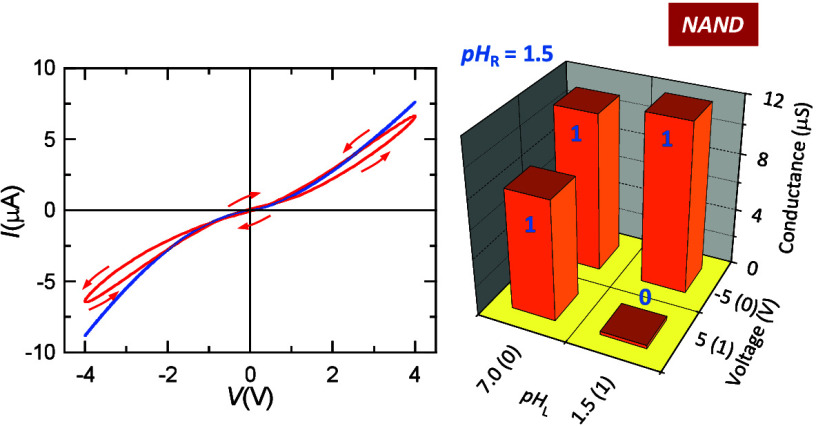

Multipore membranes with nanofluidic diodes show memristive and current rectifying effects that can be controlled by the nanostructure asymmetry and ionic solution characteristics in addition to the frequency and amplitude of the electrical driving signal. Here, we show that the electrical conduction phenomena, which are modulated by the interaction between the pore surface charges and the solution mobile ions, allow for a pH-dependent neuromorphic-like potentiation of the membrane conductance by voltage pulses. Also, we demonstrate that arrangements of memristors can be employed in the design of electrochemical circuits for implementing logic functions and information processing in iontronics.

The protein ion channels in the cell membrane allow for the transfer of matter and information in biological networks.^1^ While a wide variety of transporters can exist in the membrane, voltage-gated channels are crucial to cell bioelectricity because they may influence the counteracting dynamics of many physiological functions, including pacemaking, neural slow-wave oscillations, circadian clocks, and bioelectrical oscillatory phenomena in artificial tissues.^1,2^ The channel bioelectrical characteristics are determined by the interaction of the mobile ions in solution with the fixed charges on the pore surface.^2^ In turn, the ionization state of these charges depends upon the characteristics of the ionic solution. These facts suggest that the functionalities of biomimetic pores can be tuned by the application of both electrical and chemical signals.^3−7^ In these artificial nanopores, the control of the pore geometry and surface chemical functionalization is also possible, thus offering versatile control of the surface charge-regulated ionic transport. The above characteristics are crucial for the single-pore and multipore membranes to be employed in energy storage, water desalination, nanofiltration, biomolecule detection, and drug-controlled release.^3,4,8−11^

Memristive devices are characterized by electrical resistance that depends upon the history of applied voltages and currents. These devices can store and process information and, in the case of memristive pores,^3,4,12,13^ show a variety of surface phenomena that are biomimetic to those observed in membrane ion channels.^1,2,14,15^ We have recently described a multipore memristor with conical nanofluidic diodes^12,16^ obtained by means of track-etching techniques. The surface carboxylic acid groups show different pH-dependent ionization states, and the nanopores display distinct current–voltage curves. Taking advantage of this physical characteristic, we show that a broad range of nanopore responses can be obtained by changing the electrolyte concentration and pH of the ionic solution together with the membrane asymmetry.^17−27^ The physical insights provided suggest new functionalities based on the pH-dependent current rectification and memristive properties. In addition, arrangements of two memristors are also tuned by the pH of the solution, so that multipore membranes can be used as basic components in electrochemical circuits for signal conversion and information processing in iontronics. Potential applications concern the implementation of logic functions and controlled release processes,^3,4,8,10,28−31^ which are based on the tuning of the electrical double layer on the pore surface.^6,7,32^ The general characteristic of these pores can be found elsewhere.^10,33−35^

The *I*–*V* curves of panels a–d of Figure 1 show robust memristive characteristics^12,15,36^ that are reminiscent of those observed in biological ion channels.^1,2,14,15^ These curves were obtained for two different membrane samples to emphasize system reproducibility. The pH-regulated current rectification and pore memory effects are due to the membrane asymmetry and the shape of the pore tip, which modulates ionic conduction because of its nanoscale dimensions.^32,33^ The axial profile of the electric potential is strongly nonlinear in each pore tip because of the high volume concentration of fixed charges.^32^ For neutral pH (panels a and b of Figure 1), the pore surface charge is due to deprotonated carboxylic acid chains, displaying surface densities in the range between −0.1 and −1.0 *e*/nm^2^, where *e* is the elementary charge.^33,35^ For low enough pH values (panels c and d of Figure 1) however, the pore charge changes to positive values.^33^

**Figure 1:**
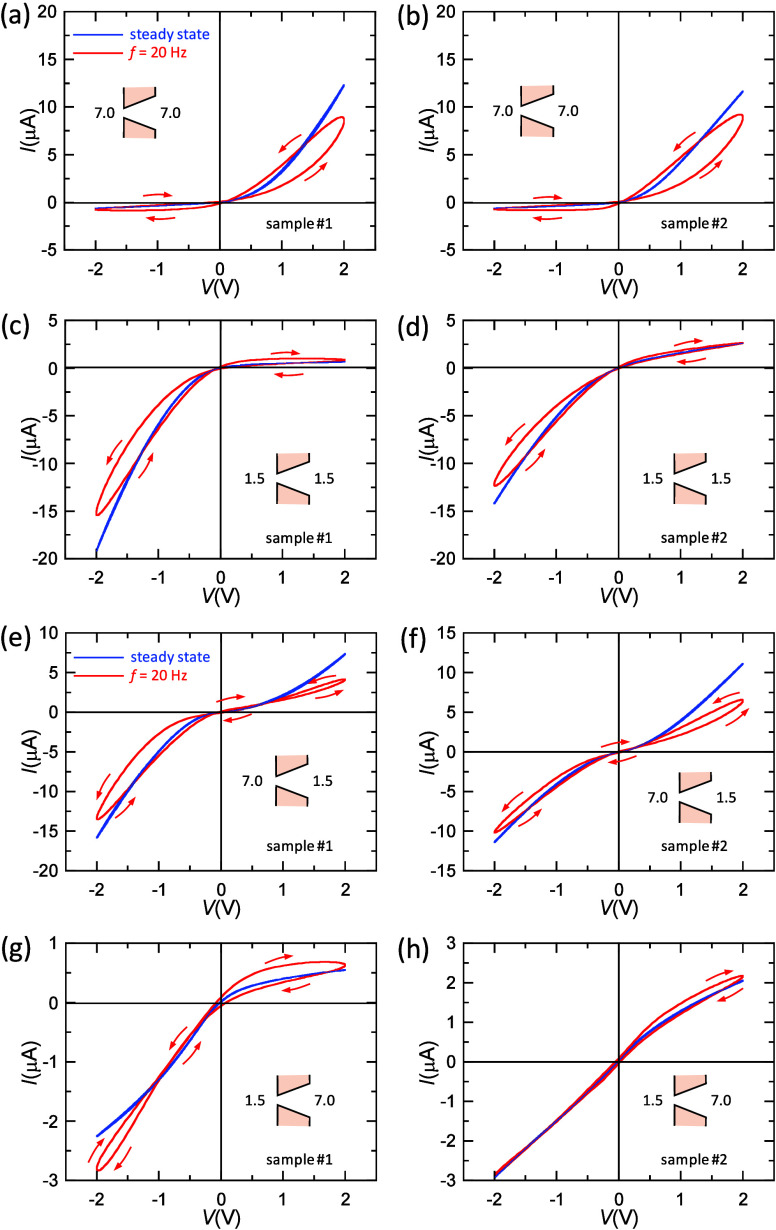
(a) Current (*I*)–voltage (*V*) curve (red) for membrane sample 1. The arrows show the time evolution of *I* as a response to a 20 Hz sinusoidal *V*(*t*) voltage bias. The time *t* evolution of the red curves indicated by arrows then corresponds to a 50 ms period signal. The steady-state *I*–*V* curve (blue) is also shown. The left and right solution pH values are pH 7.0 (tip)|pH 7.0 (base). (b) *I*–*V* curves of membrane sample 2 for the pH arrangement 7.0 (tip)|7.0 (base). (c) *I*–*V* curves of membrane sample 1 for the arrangement 1.5 (tip)|1.5 (base). (d) *I*–*V* curves of membrane sample 2 for the arrangement 1.5 (tip)|1.5 (base). (e) *I*–*V* curves of membrane sample 1 in the pH configuration 7.0 (tip)|1.5 (base). (f) *I*–*V* curves of membrane sample 2 in the pH configuration 7.0 (tip)|1.5 (base). (g) *I*–*V* curves of membrane sample 1 in the configuration 1.5 (tip)|7.0 (base). (h) *I*–*V* curves of membrane sample 2 in the pH configuration 1.5 (tip)|7.0 (base). In each case, the steady-state (blue) and 20 Hz (red) curves are shown. The insets show the particular pH configuration imposed for each membrane sample.

In negatively charged pores, at voltages *V* > 0, the solution cations accumulate at the cone tip, which gives a high pore conductance. On the contrary, at *V* < 0, these ions are depleted at the cone tip, which gives a low pore conductance. For the positively charged pores, the current rectification is reversed.^33^ The memristive behavior shows chemical inductance characteristics^37^ that arise when the ionic solution at the cone tip cannot instantaneously follow the time change of the external driving signal.^12^ As expected, the hysteretic effects are more noticeable at *V* > 0 than at *V* < 0 for the negatively charged pore (panels a and b of Figure 1) because ion accumulation occurs only in the first case. The opposite memory effects are observed for the positively charged pore (panels c and d of Figure 1) because ion accumulation occurs then at *V* < 0. The shift observed in the non-zero crossing points of the *I*–*V* curves is due to the small but non-zero capacitive current contribution, as explained previously^38^ The steady-state curves of panels a–d of Figure 1, which are obtained at a sufficiently low-frequency signal, show no hysteresis because the redistribution of the ions in the pore solution occurs on a time scale much lower than the driving signal period.^12^

Panels a–d of Figure 1 suggest that the memristive pore behavior can be modulated further using different pH values in the external solutions, which gives an additional switching control besides the driving signal characteristics. Panels e–h of Figure 1 show that this is indeed the case: different pH configurations lead to distinct current rectification and memristive effects. The distinct current rectifications obtained are primarily caused by the changes in the sign of the pore surface charges, as described in detail previously;^1,5,8,19,20,22−24,30−33^ see in particular ref (25) for a microscopic Monte Carlo study of the ionic current through a nanopore that is tuned by the external pH. Also, the different quantitative behavior observed in panels g and h of Figure 1 arises from the distinct electrical characteristics obtained in panels a–d of Figure 1 for the two membrane samples. Note however that the basic qualitative features are preserved in the two cases, as required for practical application.

Panels a–d of Figure 2 demonstrate that the current versus time curves can display neuromorphic-like spikes, which mimic neurobiological features that are present in the nervous system. The current values show synaptic potentiation in the side where the loop is inductive, and they show depression in the voltage side of capacitive loops of Figure 1. This result is potentially useful for computing because trains of sequences with positive and negative square-wave voltage pulses can be applied to the membrane.^16^ The different pH configurations imposed here will then give distinct conductance changes with time. These pH-modulated conductances, obtained as responses to different voltage pulses, can offer an alternative to the traditional steady-state current–voltage curves for implementing logic functions, as we will show later using different sets of electrochemical inputs.

**Figure 2:**
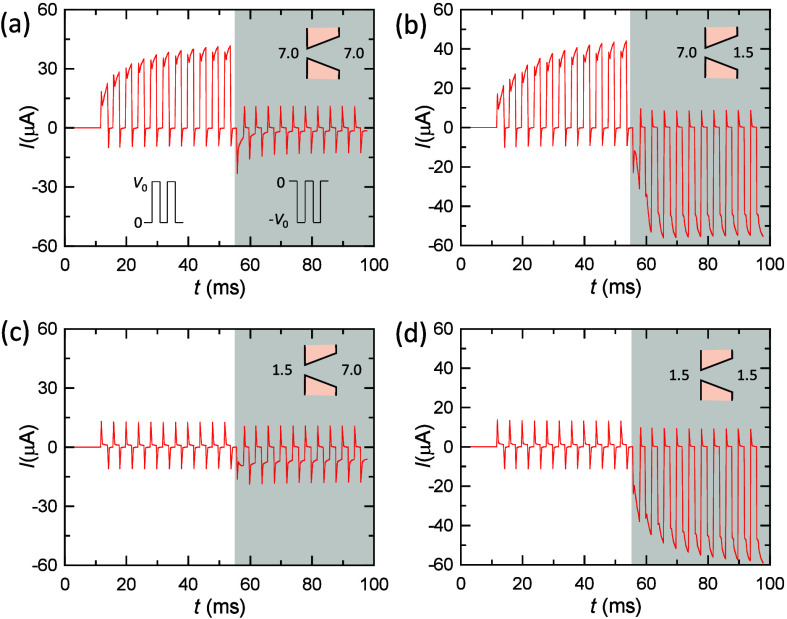
(a) *I* versus time curves for sample 1. The current spikes are obtained using sequences of 11 positive and 11 negative voltage pulses with amplitude *V*_0_ = 5 V, 2 ms duration, and 2 ms interval. The pH configuration 7.0 (tip)|7.0 (base) gives a gradual conductance increase for positive amplitude and a low conductance state for negative amplitude. (b) pH configuration 7.0 (tip)|1.5 (base) gives gradual conductance increases for positive and negative amplitudes. (c) pH configuration 1.5 (tip)|7.0 (base) gives low conductance states for positive and negative amplitudes. (d) pH configuration 1.5 (tip)|1.5 (base) gives a low conductance state for positive amplitude and a gradual conductance increase for negative amplitude.

Panels a–h of Figure 3 show the *I*–*V* curves obtained for series arrangements of membrane samples 1 and 2, which allow for different pH configurations in the left, central, and right solutions. In particular, the pH in the central solution can be equal to or different from that of the left and right solutions. To concentrate our study on the pH effects, we have considered only symmetric arrangements, with the two membranes facing either the pore tips or the base tips and the same pH in the left and right solutions. However, the resulting *I*–*V* curves can still be asymmetric in panels d, e, g, and h of Figure 3 because of the different rectification properties of samples 1 and 2 used in the distinct pH configurations. Note here that, when the two membranes are combined in the same series pH configuration, the particular order of identical samples should be irrelevant for the *I*–*V* curves, as approximately shown by the curve pairs of panels a and b, panels c and f, panels d and e, and panels g and h of Figure 3. However, the different individual membrane characteristics (Figure 1) can give current rectifications in some pH configurations (Figure 3).

**Figure 3:**
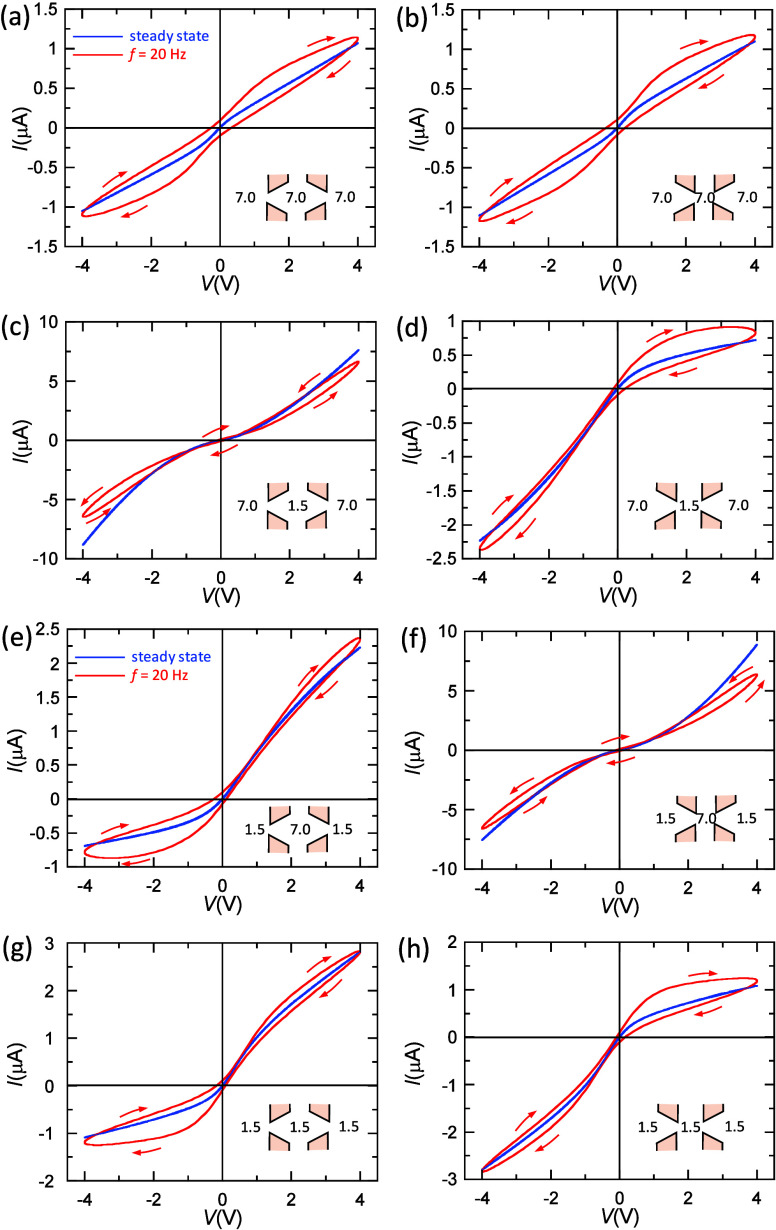
(a) *I*–*V* curves for membrane samples 1 and 2 in a series arrangement with the following left, central, and right pH configuration 7.0 (tip)|7.0 (base–base)|7.0 (tip). (b) *I*–*V* curves for the pH configuration 7.0 (base)|7.0 (tip–tip)|7.0 (base). (c) *I*–*V* curves for the pH configuration 7.0 (tip)|1.5 (base–base)|7.0 (tip). (d) *I*–*V* curves for the pH configuration 7.0 (base)|1.5 (tip–tip)|7.0 (base). (e) *I*–*V* curves for the pH configuration 1.5 (tip)|7.0 (base–base)|1.5 (tip). (f) *I*–*V* curves for the pH configuration 1.5 (base)|7.0 (tip–tip)|1.5 (base). (g) *I*–*V* curves for the pH configuration 1.5 (tip)|1.5 (base–base)|1.5 (tip). (h) *I*–*V* curves for the pH configuration 1.5 (base)|1.5 (tip–tip)|1.5 (base).

Panels a–h of Figure 4 show the current versus time curves corresponding to the tip|base–base|tip and base|tip–tip|base series of pH configurations, respectively. As in Figure 2, the current spikes are obtained using 2 ms sequences of positive and negative voltages. Taking together, the results suggest that virtually universal responses can be obtained by changing the relative orientations of the external pH difference and the pore position gradient in the different pH configurations of the series arrangement. The current rectification and memristive functionalities obtained provide a complete catalogue of on/off conductance states, inward/outward rectifications, and memory effects that mimic those observed in voltage-gated ion channels under different biological conditions.^2,14,15,21^ We highlight the remarkable result of synaptic potentiation in both positive and negative voltage in panel c of Figure 4, corresponding to double inductive loops in panel c of Figure 3.

**Figure 4:**
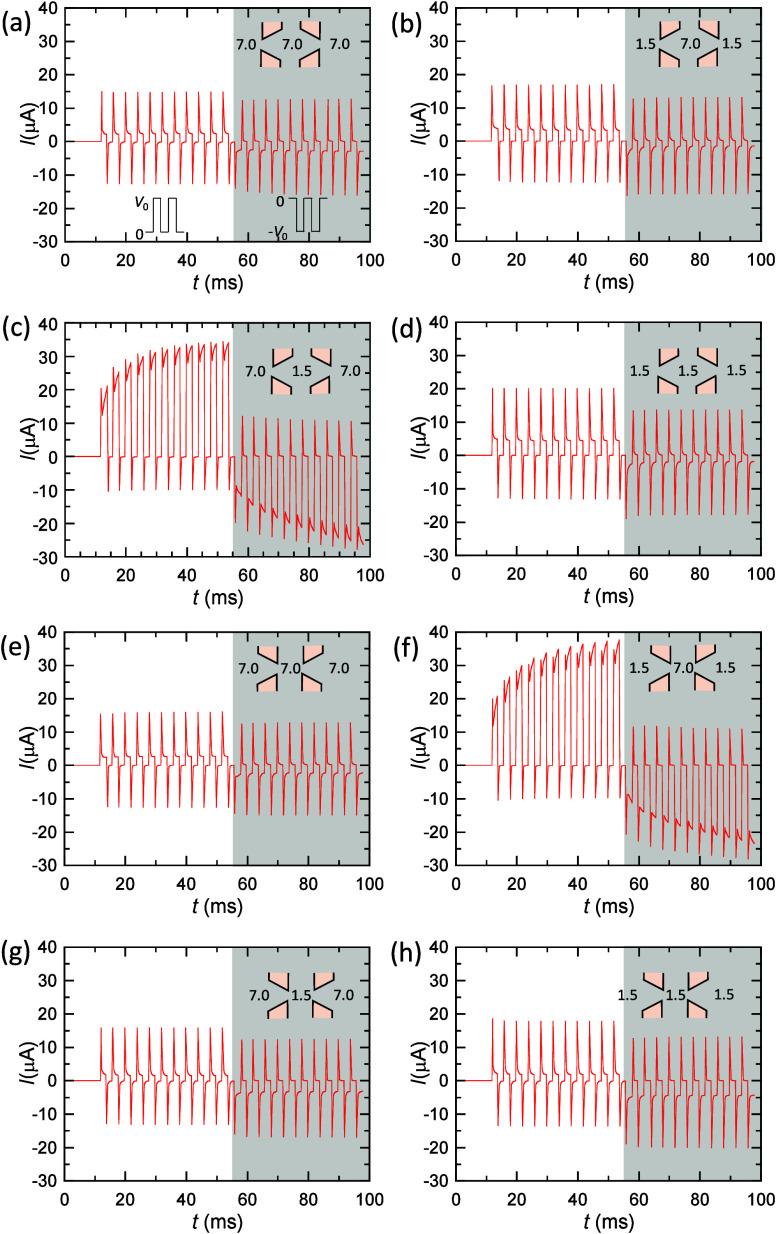
(a) *I* versus time curves for membrane samples 1 and 2 in the series arrangement for the pH configuration 7.0 (tip)|7.0 (base–base)|7.0 (tip). The current spikes are obtained using the above sequences of positive and negative voltage pulses. (b) *I* versus time curves for the pH configuration 1.5 (tip)|7.0 (base–base)|1.5 (tip). (c) *I* versus time curves for the pH configuration 7.0 (tip)|1.5 (base–base)|7.0 (tip). (d) *I* versus time curves for the pH configuration 1.5 (tip)|1.5 (base–base)|1.5 (tip). (e) *I* versus time curves for the series arrangement of membrane samples 1 and 2 in the pH configuration 7.0 (base)|7.0 (tip–tip)|7.0 (base). (f) *I* versus time curves for the pH configuration 1.5 (base)|7.0 (tip–tip)|1.5 (base). (g) *I* versus time for the pH configuration 7.0 (base)|1.5 (tip–tip)|7.0 (base). (h) *I* versus time for the pH configuration 1.5 (base)|1.5 (tip–tip)|1.5 (base). The current spikes are obtained using sequences of 11 positive and 11 negative voltage pulses with amplitude *V*_0_ = 10 V, 2 ms duration, and 2 ms interval.

We aim now at showing how the memristive pore could be used as logical physicochemical devices. To this end, Figure 5 shows the different logic responses that can be obtained using the solution pH and the applied voltage *V* as the input variables together with the steady-state membrane conductance *G* = *I*/*V* as the output variable. As a proof of concept, the OR and INHIBIT-1 and INHIBIT-2 (inhibit) functions, together with the universal NAND function, are shown, which suggests potential applications in iontronic circuits.^3,4,28,29,39,40^ As an alternative to the above steady-state logics, unconventional neuromorphic computing based on pH-modulated conductance potentiation could also be implemented (Figures 2–4). Note here that the solution pH and applied voltage are common variables in most electrochemical devices.

**Figure 5:**
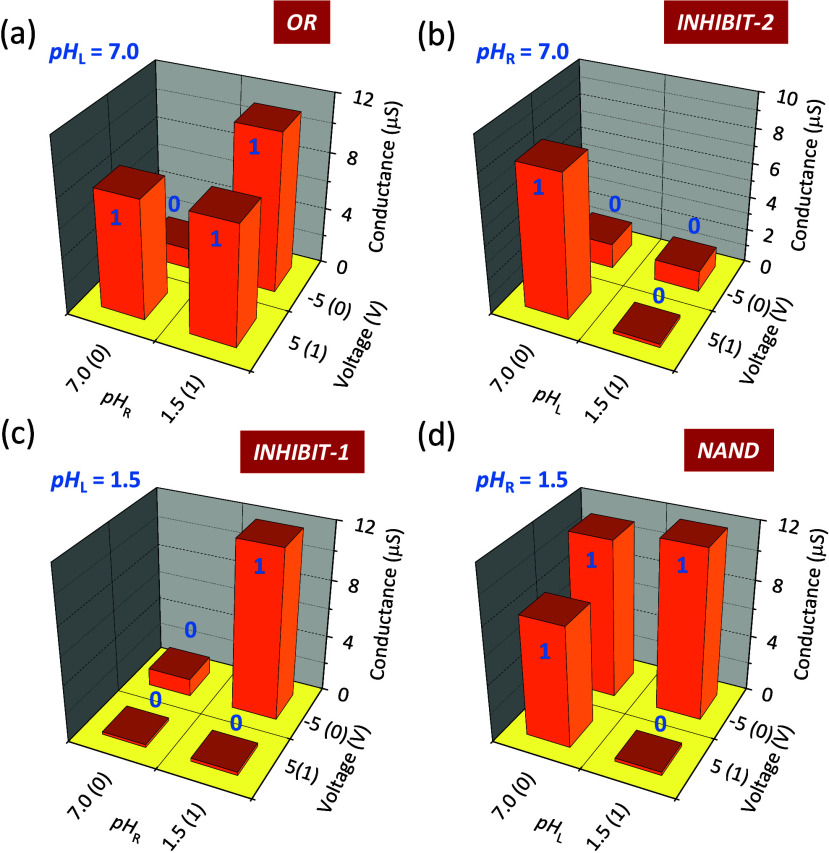
Logic function responses obtained by fixing either pH_L_ (left solution) or pH_R_ (right solution) in the case of sample 1. The input 1 variable is the sign of the applied voltage *V* (0 for *V* = −5 V and 1 for *V* = 5 V). The input 2 variable is the non-fixed solution pH (0 for pH 7.0 and 1 for pH 1.5). The output variable is the steady-state membrane conductance *G* = *I*/*V* (0 for low *G* and 1 for high *G*). The logical responses correspond to the (a) OR, (b) INHIBIT-2, (c) INHIBIT-1, and (d) universal NAND functions.

Surface charge-modulated ionic transport in soft nanostructures is central to current materials science and technology. Multipore membranes with nanofluidic diodes display pH-dependent memristive and current-rectifying characteristics that are determined by the interaction between the pore surface charges and the solution mobile ions. Thus, they can be controlled by the nanostructure asymmetry and the ionic solution characteristics in addition to the frequency and amplitude of the electrical driving signal. The memristive effects observed allow for a neuromorphic-like potentiation of the membrane conductance that is regulated by voltage pulses. Also, we have suggested that arrangements of memristive membranes could be employed in the design of electrochemical circuits for implementing logic functions and information processing in iontronics.^4,39−42^

## Methods

The current (*I*)–voltage (*V*) curves of the nanofluidic memristor were measured with 50 mM KCl aqueous solutions at pH 7.0 (negatively charged pore) and pH 1.5 (positively charged pore).^12^ To this end, a sinusoidal wave of potential amplitude *V*_0_ = 2 V and frequency *f* = 20 Hz was used. The steady-state *I*–*V* curves corresponding to a low-frequency (25 mHz) signal were also measured. The multipore membrane design and preparation have been described previously.^12^ The irradiation of 12.5 μm thick polyimide foils by swift heavy ions and the subsequent functionalization of the resulting tracks by means of asymmetric track-etching techniques^10,33−35^ produced a multipore membrane with conical nanopores whose typical tip and base radii were of the order of 10 and 100 nm, respectively.^33^ The multipore membrane exposed area was approximately 1 cm^2^. All electrical measurements were made using a homemade electrochemical cell with Ag|AgCl electrodes connected to a BioLogic SP-200 potentiostat. The cell was placed inside a magnetic shield on an antivibration table. Good data reproducibility was found, as described in detail previously.^12,33^
